# Hyde on the Skin

**Published:** 1900-06

**Authors:** 


Hyde on the Skin.—New (5th) edition. A practical treatise on diseases of the skin, for the use of students and practitioners, by James Nevins Hyde, A. M., M. D., Professor of Dermatology and Venereal Diseases in Rusk Medical College, Chicago. In one octavo volume of 866 pages, with 111 engravings and 24 full-page plates, eight of which are colored. Cloth, $4.50, net; leather, $5.50, net. Lea Brothers & Co., Philadelphia and New York. 1900.

This is one of the most popular books on the diseases of the skin. Since the first edition about seventeen years ago, the work has undergone five thorough and complete revisions. To the last were added forty-eight pages and twelve full-page plates. It has been so long before the profession that it has become the text-book in nearly all the colleges, and is perhaps in more private libraries than any book on the subject. Its great popularity is due mainly to the fine arrangement of the subject matter and to its thorough practicability.

